# *E*/*Z* isomerization of astaxanthin and its monoesters in vitro under the exposure to light or heat and in overilluminated *Haematococcus pluvialis* cells

**DOI:** 10.1186/s40643-021-00410-5

**Published:** 2021-07-01

**Authors:** Yauhen V. Viazau, Ruslan G. Goncharik, Irina S. Kulikova, Evgeny A. Kulikov, Raif G. Vasilov, Alla A. Selishcheva

**Affiliations:** 1 Institute of Biophysics and Cell Engineering, National Academy of Sciences of Belarus, Akademicheskaya St. 27, 220072 Minsk, Belarus; 2 National Research Center Kurchatov Institute, Akademika Kurchatova Sq. 1, Moscow, 123182 Russia; 3 Lomonosov Moscow State University, Leninskie gory 1, Moscow, 119991 Russia

**Keywords:** Astaxanthin, *Haematococcus pluvialis*, *E*/*Z* isomerization, HPLC

## Abstract

Thermo- and photoisomerization of astaxanthin was investigated in a model system (solutions in methanol and chloroform), and the dynamics of astaxanthin isomers and esters content was analyzed in *Haematococcus pluvialis* green algal cells exposed to factors inducing astaxanthin accumulation. In both systems, the astaxanthin isomerization process seems to be defined by a) the action of light (or heat), and b) the dielectric constant of the surrounding medium. Upon heating, the accumulation of *Z*-isomers occurred in a model system during the entire incubation period. For the first 5 h of illumination, both *Z*-isomers accumulated in the solutions up to 5%, and then their content decreased. The accumulated amount of the *Z*-isomers in the cells of *H. pluvialis* was found to reach 42% of the total content of astaxanthin initially, and then it decreased during the experiment. The results lead to a conclusion that both cultivation of *H. pluvialis* culture in specific conditions and heat treatment of the resulting extracts from it might be efficient for obtaining large amounts of economically useful astaxanthin *Z*-isomer.

**Supplementary Information:**

The online version contains supplementary material available at 10.1186/s40643-021-00410-5.

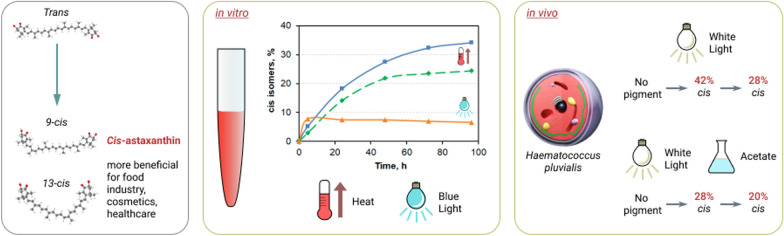

## Introduction

**Fig. 1:**
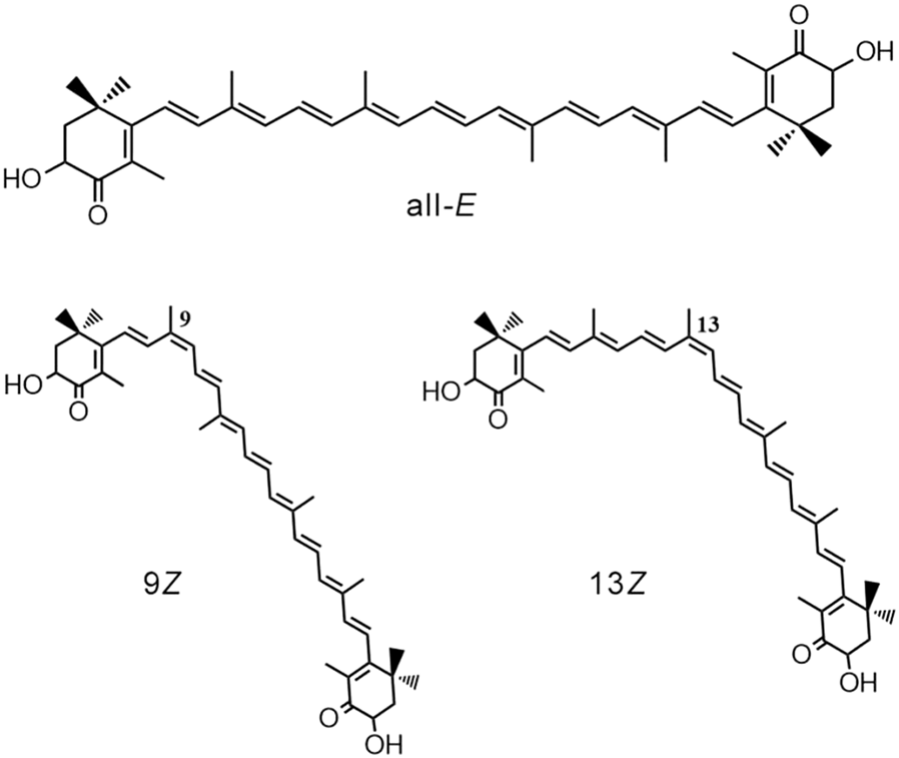
Geometric isomers of astaxanthin

The molecule of astaxanthin, a xanthophyll pigment, contains two hydroxyl and two carbonyl groups as well as a long polyene carbon chain; therefore, it can exist in the form of several geometric (Fig. 1) and optical isomers.

Potential health benefits of astaxanthin have been extensively studied and there is a large amount of evidence for health effects of this pigment (Guerin et al. 2003; Higuera-Ciapara et al. 2006; Hussein et al. 2006; Kidd 2011; Fakhri et al. 2018). For astaxanthin, anti-apoptotic, anti-aging, anti-cancer, anti-obesity, cardioprotective, immuno-modulatory, anti-diabetic, hepatoprotective and neuroprotective activities were described, as well as its positive effects on skin, eye retina, sports performance, reproduction system (Fakhri et al. 2018). For example, the substantial body of clinical studies examining the effects of astaxanthin dietary supplementation on human skin health showed the improvement in skin texture, appearance and moisture content after 2‒16 weeks of treatment (Ng et al. 2021).

In nature, astaxanthin is mostly represented by the all-*E*-isomer, where the carbons at all the double bonds are located in the *E* positions with respect to each other. However, all-*E*-astaxanthin can undergo isomerization, forming a mixture of the 9*Z*- and 13*Z*-isomers (Yuan and Chen 1999), when affected by many factors, such as solvents, temperature, metal ions, or pH of the reaction medium (Zhao et al. 2005; Chen et al. 2007; Lerfall and Birkeland 2014; Honda et al. 2019a). It has been found that the isomerization reaction is reversible (Yuan and Chen 2001); therefore, the less stable *Z*-isomers are gradually converted to more stable all-*E* configuration. Unfortunately, there are a very few studies concerning the effect of light on the isomerization process, although it is under the exposure to intense illumination that astaxanthin and its esters are synthesized by the microalgae, and thus such studies should be regarded as necessary. Photoisomerization of astaxanthin has been studied both in the *E*/*Z* isomerization reaction (Weesepoel et al. 2014; de Bruijn et al. 2016) and in the reverse *Z*/*E* isomerization reaction (Honda et al. 2020) practically under the same conditions: at high temperature and light source radiation power of 30‒40 W. Different results were obtained for each type of reaction: for the direct reaction, not only the resulting *Z*-isomers were identified, but also the products of oxidative destruction of astaxanthin, while as for *Z*/*E* isomerization, data on the astaxanthin oxidative destruction is absent.

The interest to astaxanthin *Z*-isomers is due to their higher antioxidant properties and health benefits compared to all-*E*-astaxanthin, and enhanced bioavailability. It was shown that 13*Z*-astaxanthin selectively accumulates in human plasma (Østerlie et al. 2000; Yang et al. 2017a). *E*/*Z* isomerization improves the efficiency of processing (extraction, emulsification, encapsulation) and bioavailability of astaxanthin and carotenoids in general (Honda et al. 2019a, b; Honda 2020). Higher *Z-*isomer ratios of up to 50% in astaxanthin feed supplements for laying hens led to a fivefold increase in astaxanthin concentration in egg yolk, twofold increase in yolk color fan score and fivefold increase in astaxanthin content in blood plasma of hens compared to all-*E*-astaxanthin supplement (Honda et al. 2021).

Liu and Osawa have shown that in both stable radical DPPH scavenging activity test and in rat microsome and rabbit erythrocyte ghost membrane lipid peroxidation systems, *Z*-astaxanthin, and especially 9*Z*-astaxanthin, exhibits a stronger antioxidant effect than the all-*E*-isomer (Liu and Osawa 2007). In addition, 9*Z*-astaxanthin was shown to be the most effective among the astaxanthin isomers for inhibition of the generation of reactive oxygen species in human neuroblastoma cells as well as for the inhibition of collagen type II degradation induced by hydroperoxides (Liu and Osawa 2007). It was also shown that 13*Z*-astaxanthin has stronger antioxidant activity compared to all-*E* and 9*Z* in oxygen radical absorbing capacity assay for lipophilic compounds, photochemiluminescence and cellular antioxidant activity assay (Yang et al. 2017b). Analysis of the ability of astaxanthin isomers to scavenge DPPH free radicals, ABTS radicals and superoxide anions showed that *Z*-isomers were more effective in this regard than all-*E*-astaxanthin (Jin et al. 2010).

Several studies in recent years have shown that *Z*-astaxanthin is more beneficial for human and animal health. It was reported that while all three astaxanthin isomers extended the lifespan of *C. elegans* worm, 9*Z*-astaxanthin was the most effective for this purpose (Liu et al. 2018). *Z*-astaxanthins, and especially 9*Z*-astaxanthin, exhibited greater anti-inflammatory effect than all-*E*-astaxanthin by down-regulating pro-inflammatory cytokines IL-8 and COX-2 as well as TNF-α gene expression in Caco-2 cell monolayer model. Inhibition of specific transcription factors such as NF-κB could be the mechanism behind astaxanthin anti-inflammatory effect. Interestingly, the same authors reported that 13*Z*-astaxanthin had significantly higher affinity with scavenger receptor class B type I transporter protein than other isomers, which can at least partially explain its higher bioavailability (Yang et al. 2017a, 2019). Such data makes *Z*-astaxanthin a good candidate for anti-inflammatory agent in treatment of human diseases associated with bowel epithelium inflammation.

A significant amount of astaxanthin, mostly in the esterified form, can accumulate under the action of various stress factors in the cells of green alga *Haematococcus pluvialis* (Kakizono et al. 1992; He et al. 2018). In addition to the exposure to high-intensity light (including the initiation of the photo-oxidative stress), the accumulation of astaxanthin and its esters can be induced by the UV irradiation, deficiency or excess of biogenes, or salinization of the medium (Boussiba and Vonshak 1991; Sarada et al. 2002; Zhekisheva et al. 2002). For example, it has been shown that the accumulation of astaxanthin and its esters can occur under the exposure to high concentrations of CO_2_, which lead to an increase in the C/N ratio and, consequently, could be percepted by the cell as nitrogen deficiency (Christian et al. 2018). When sodium acetate, divalent iron ions, or sodium chloride were added to the culture medium, causing a salt stress (Kobayashi 1993; He et al. 2018), the content of this carotenoid was shown to increase as well, and the expression of the key genes of its biosynthesis pathway was shown to be activated (Ota et al. 2018; Zhang et al. 2019). The cells of *H. pluvialis* can contain up to 3–5% astaxanthin in their dry matter (Johnson and Schroeder 2006); thus, this alga is one of the most promising sources of this pigment, which is used as a bioactive supplement in food, cosmetics, pharmaceuticals, and also as a livestock food supplement. Unfortunately, most studies concerning this topic investigate the total content of astaxanthin (Kakizono et al. 1992; Kobayashi 1993; Christian et al. 2018), ignoring the contents and functions of the mono- and diesters.

The study of the accumulation of individual astaxanthin isomers in *H. pluvialis* cells exposed to stressful conditions can shed a light on differences and similarities in astaxanthin isomerization in a model system and in a living cell, as well as it can help to evaluate the prospects for obtaining algal biomass enriched with *Z*-isomers. Such study should include the analysis of astaxanthin esters content and composition as astaxanthin is mainly accumulated in esterified form in *H. pluvialis* cells, which can directly affect the process of isomerization.

The aim of our research was to investigate the thermo- and photoisomerization (*E/Z* isomerization) of astaxanthin in a model system (solutions in methanol and chloroform), and to analyze the dynamics of astaxanthin isomers and esters content in *Haematococcus* cells exposed to factors inducing astaxanthin accumulation.

## Materials and methods

### Reagents

Hexane for high-performance liquid chromatography (HPLC), acetonitrile for HPLC, methanol for HPLC, ethyl acetate for HPLC, triethylamine, all-*E*-astaxanthin (97%, HPLC-grade) (Sigma-Aldrich, USA). Deionized water Milli-Q 18.2 MΩ·cm was obtained using a Millipore Integral 10 purification system.

### Identification of the all*-E-* and *Z-*isomers of astaxanthin using HPLC with mass detection (HPLC–MS) and photometric detection in the visible range

The identification of the free astaxanthin isomers was carried out using HPLC using an Agilent 1200 series liquid chromatograph with a diode array detector on a ZORBAX Eclipse XDB-C18 reversed-phase column (250 × 4.6 mm, particle size 5 μm) according to the method described in (Yuan and Chen 1999). The detection was carried out at a wavelength of 480 nm. The mobile phase was represented by the following system: MeOH: CH_2_Cl_2_: CH_3_CN: H_2_O = 82.5: 5: 5.5: 7. The mode was isocratic during the entire test. Flow rate 800 μl/min, injection volume 40 μl, temperature 30 °C. Isomers were qualitatively determined from their absorption spectra in the visible region.

HPLC–MS was conducted in the positive ion mode within the 0‒1000 m/z mass-to-charge range. All the samples introduced into the chromatographic column were dissolved in methanol. To calculate the concentrations of the all-*E*-, 9*Z*-, and 13*Z*-isomers in the samples, we used the extinction coefficients 125,100, 158,000, and 88,100 l/mol∙cm, respectively (de Bruijn et al. 2016).

The most intense peaks correspond to all-*E*- *(1)*, 13*Z*- *(2)* and 9*Z*-isomers (*3*) (see Additional file 1). In the later stages of incubation, lower peaks for other *Z*-isomers, which are formed in solution in trace amounts, may also appear. The absorption maximum for peak 1 corresponds to the known values of the all-*E*-isomer and is at 480 nm. Peaks 2 and 3 were identified as 13*Z*- and 9*Z*-isomers, respectively, by the characteristic shift of the absorption maximum to the region with shorter wavelengths (for the 9*Z*-isomer—472 nm, for the 13*Z*-isomer—468 nm), as well as the appearance of a second absorption maximum for the 13*Z*-isomer (370 nm). Mass spectrometry showed that the same ion with m/z equal to 597.3, which is characteristic of the free form of astaxanthin, corresponds to peaks 1–3. This fact confirms the conclusion that peaks 1‒3 are isomers of the same compound.

### The formation of astaxanthin *Z*-isomers during prolonged incubation at 40 °C

A stock solution of of all-*E*-astaxanthin (identified by HPLC) in methanol or chloroform (0.01 mg/ml) with a volume of 2 ml was incubated for 7 days at 40 °C. After 5 h of incubation, as well as every 24 h throughout the entire experiment, a 200 μl sample was taken to determine the amount of isomers by HPLC. Before injection, the samples were preliminarily redissolved in a mixture used as a mobile phase.

### The formation of the *Z*-isomers and the degradation of astaxanthin exposed to blue light at 25 °C

The stock solution of free astaxanthin in methanol (0.01 mg/ml) with a volume of 2 ml was divided into two 1 ml samples. The control sample was stored in a refrigerator (5 °C) without exposure to light for 4 days, and the test sample was irradiated with blue light (450–500 nm) at an illuminance of 28,000 lx at room temperature (25 °C) also for 4 days. The optical densities of the solutions in question were measured with a Varian UV–Vis Cary 50 spectrophotometer once a day, and the contents of the *Z*-isomers were determined using HPLC according to the above method.

### *H. pluvialis* cells cultivation

The object of the research was an algologically pure culture of the unicellular flagellate green alga *H. pluvialis*, strain IBCE H-17, from the algae collection of the Institute of Biophysics and Cell Engineering, National Academy of Sciences of Belarus. The culture was passaged from the agarized BBM medium to Rudic’s liquid medium and grown for 5 days under light of normal illuminance for this alga (1500 lx) with a light period of 14 h and a dark period of 10 h, temperature in the light period 23 ± 1 °C.

Then the culture was exposed to intense illumination (9000 lx) and to sodium acetate for 12 days. During both growing and stressing, the cell suspension was illuminated using fluorescent white lamps. The intensity and source of the light were chosen so that, despite the stressing effect of the light, part of the algal cells could survive and function, accumulating astaxanthin. The stressed cells were cultivated under different conditions: in the acetate variant, 1 g/l sodium acetate was added to the culture medium; in the control variant, nothing was added and the cells were stressed using light only. Thus, the accumulation of astaxanthin was induced either by high-intensity light alone or in combination with an excess of the nutrient source (sodium acetate). The number of cells per unit volume of the suspension was calculated using a Goryaev chamber and a Nikon Eclipse TS100 optical microscope. The samples for the analysis were taken before the beginning of the stress (day 0) and on the day 5 and day 12 of the experiment.

### Detection of pigments in *H. pluvialis* cells

The contents of the photosynthetic pigments in acetone extracts and of astaxanthin in methanol extracts from the *Haematococcus* cells were determined according to the methods described in (Averina et al. 2018) and recalculated per the number of cells. Total photosynthetic carotenoids were calculated as sum of neoxanthin, violaxanthin, lutein and β-carotene. To determine the isoforms of astaxanthin, we used the extracts after saponification (addition of 10 μl 1 M KOH to 0.5 ml of the extract and incubation in the dark for 6 h), and the esters were determined in the initial (unsaponified) extracts. The content of astaxanthin was calculated using commercial astaxanthin (Sigma-Aldrich, US) as the analytical standard.

### HPLC–MS determination of the fatty acid composition of astaxanthin monoesters

**Fig. 2:**
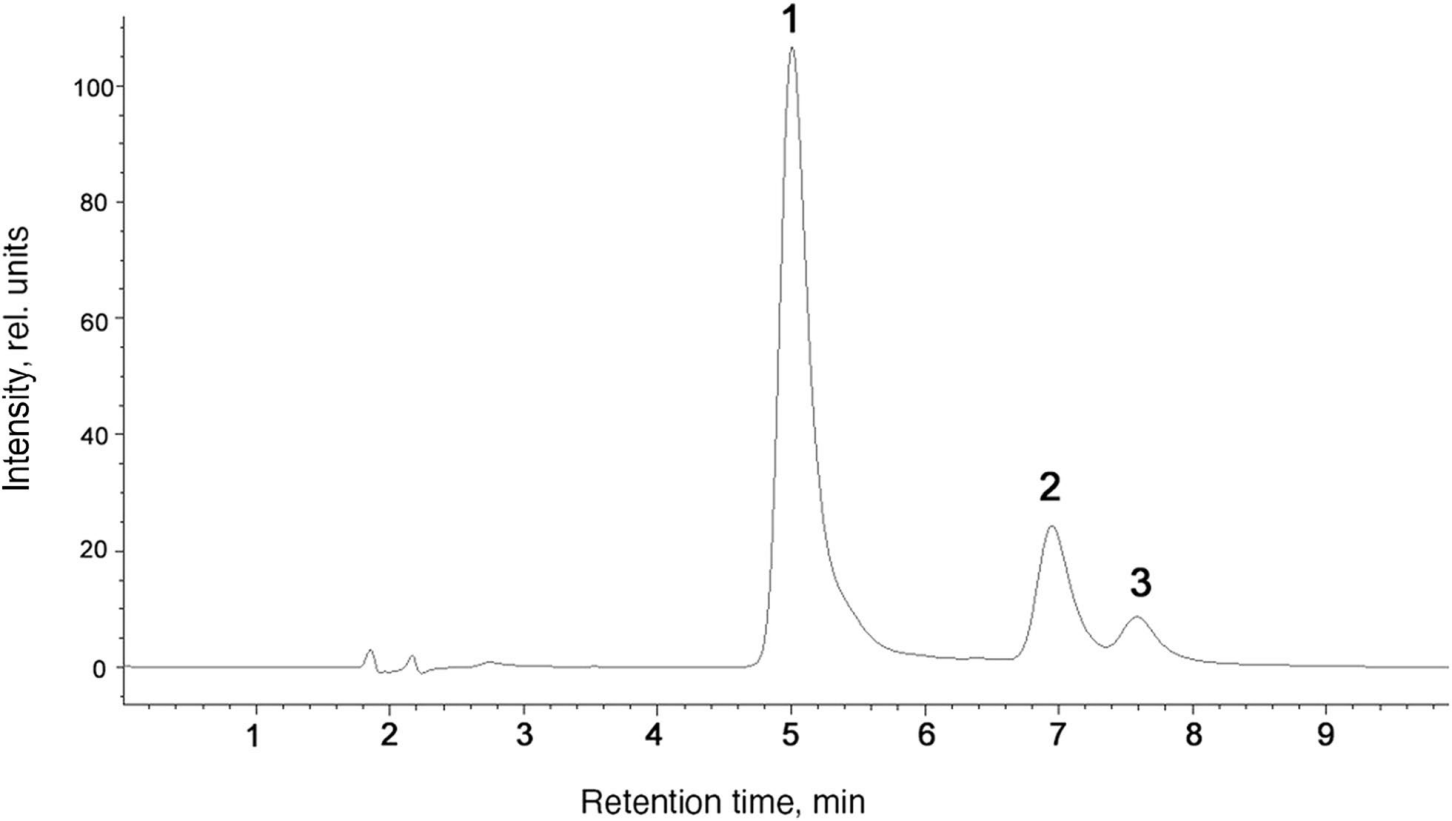
Chromatogram of astaxanthin solution after incubation in chloroform for 2 days at 40 °C: (1) all-*E*-astaxanthin, (2) 13*Z*-astaxanthin, (3) 9*Z*-astaxanthin

The *Haematococcus* cells were extracted in *n*-hexane, producing a crude extract, where astaxanthin was almost exclusively in the esterified (mostly in the monoester) form. The esters were separated using HPLC on an Agilent 1200 (US) liquid chromatograph with a diode array detector on a reversed-phase column (150 × 2.1 mm, particle size 5 μm, Orbit-C18) (Fig. 2) with the detection at 470 nm. As the mobile phases, we used 50% (vol/vol) acetonitrile in H_2_O (Millipore) (solution A) and 100% acetonitrile (solution B). Flow rate 700 μl/min, injection volume 20 μl. The elution program started from 25% A and 75% B and continued in the gradient elution mode (0‒12 min) up to 100% B; from 12 min till the end, the mode was isocratic at 100% (vol/vol) of the mobile phase B. The mass spectrometry was performed in the positive ion mode using the same chromatographic column and the same elution conditions. The injection volume was 8 μl of the astaxanthin monoesters (*c* = 0.5 mg/ml). The data were recorded in the 830–870 and 870–900 m/z ranges.

## Results

### Astaxanthin thermal and photoisomerization in model systems

In previous studies of the process of all-*E*-astaxanthin isomerization under illumination and heating in various solvents, a small percentage of *Z*-isomers was obtained, not exceeding 7–8%. The first step of the work was to select the conditions for obtaining high amount of *Z*-isomers. For this, thermal and photoisomerization experiments in model system described here were carried out for 4 days each.

**Fig. 3:**
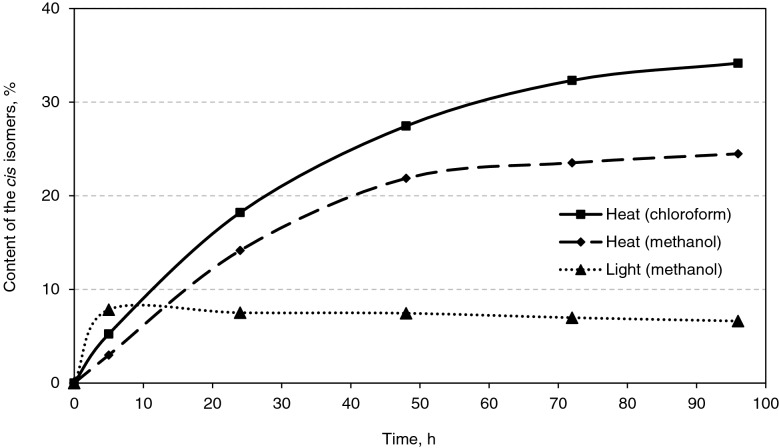
Change in the total content of astaxanthin *Z*-isomers under different conditions: (Heat)—incubation at 40 °C in the dark; (Light)—incubation at room temperature under blue light (28,000 lx)

Figure 3 shows that the rate of *Z*-isomers formation for the first 5 h of the photoisomerization experiment was much higher than upon heating. However, after 5 h of illumination, the content of *Z*-isomers began to decrease, while during thermal isomerization, its rise continued further, gradually slowing down.

It was suggested that this dynamics may be associated with the rapid destruction of astaxanthin under the action of light, while in the dark at 40 °C astaxanthin degraded rather slowly allowing for accumulation of substantial amount of *Z*-isomers. To confirm this, we compared the absorption spectra of astaxanthin after 5 h of illumination to its spectra after prolonged incubation at 40 °C and in control sample (see Supplementary material). It showed the increase of the absorption in the region of 250–260 nm, which corresponds to the absorption maximum of apo-9-astaxanthinone (de Bruijn et al. 2016). However, it was not observed on the absorption spectrum of astaxanthin after incubation for 4 days in methanol at 40 °C. Here, the spectrum had a higher absorption in the 250–400 nm region, which is most likely due to the high content of 13*Z*-isomer, which has a wide shoulder on the spectrum in this region.

## Astaxanthin and its esters in *H. pluvialis* cells subjected to various types of stress

The next stage of the research was aimed at determining the content of the *Z*-isomers in the astaxanthin fraction synthesized in *H. pluvialis* cells at different times of the exposure to stress factors such as high light intensity and addition of sodium acetate in culture medium. It is known that astaxanthin is synthesized in algal cell in form of its all-*E*-isomer. Therefore, any amount of *Z*-isomers found in the cell would be due to an isomerization process. For the purpose of exploring biotechnological applicability of the induction of astaxanthin accumulation by high light intensity combined with acetate addition to the medium, as well as to gain knowledge on the general response of *H. pluvialis* culture to such conditions, we also determined cell number and cell diameter.

### Cell survival during stress

**Fig. 4:**
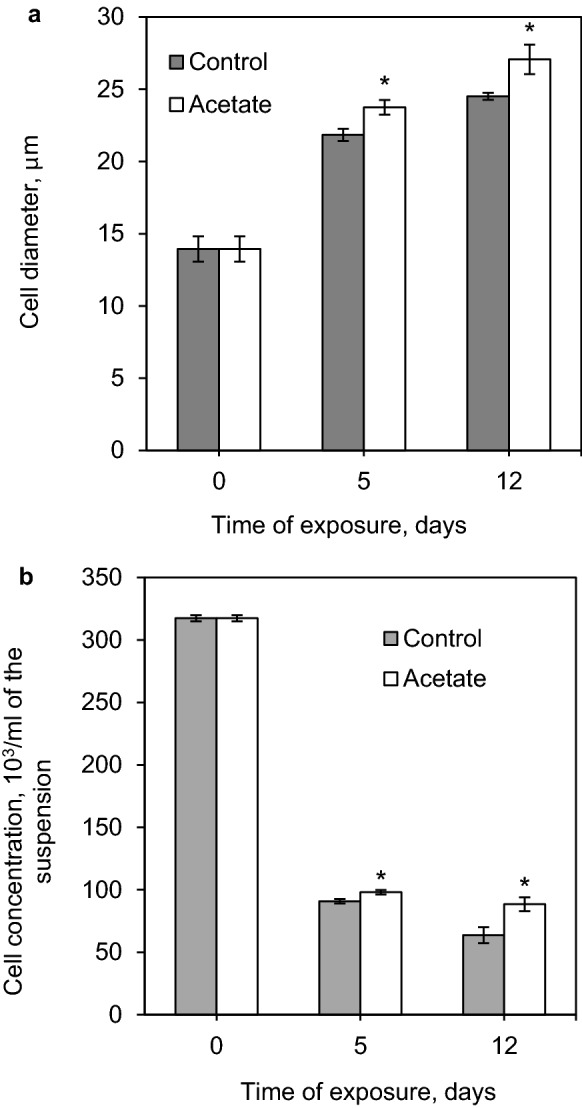
Cell (**a**) diameter and (**b**) concentration in *H. pluvialis* suspension after 5 and 12 days of cultivation under stress conditions: hereinafter (*) indicates significant difference from the corresponding control (*p * ≤  0.05)

It was found that prolonged exposure to stress causes the transformation of the *H.* *pluvialis* cells into nonmotile large cysts with an average size of 23 μm on 5 days and 26 μm on 12 days of the exposure (Fig. 4A); the initial value for the green cells (day 0) is 14 μm. The diameter of the cells in the acetate variant was 9% and 11% higher than the control value on days 5 and 12 of the exposure, respectively. The calculation of the cell concentrations in culture medium showed that the irradiation with high-intensity light caused most of the cells (up to 80%) to die (Fig. 4B). The addition of sodium acetate under stress conditions caused a 9% increase in the number of the survived cells by 5 days and a 39% increase by day 12 of the exposure to stress. Thus, the addition of sodium acetate accelerates the process of cyst formation during the exposure to high-intensity light and favors the survival of the cells under these conditions. Both effects of sodium acetate (the increase in the diameter and in the number of cells) become more prominent with time.

### Content of astaxanthin esters in *H. pluvialis* cells affected by various types of stress

**Fig. 5:**
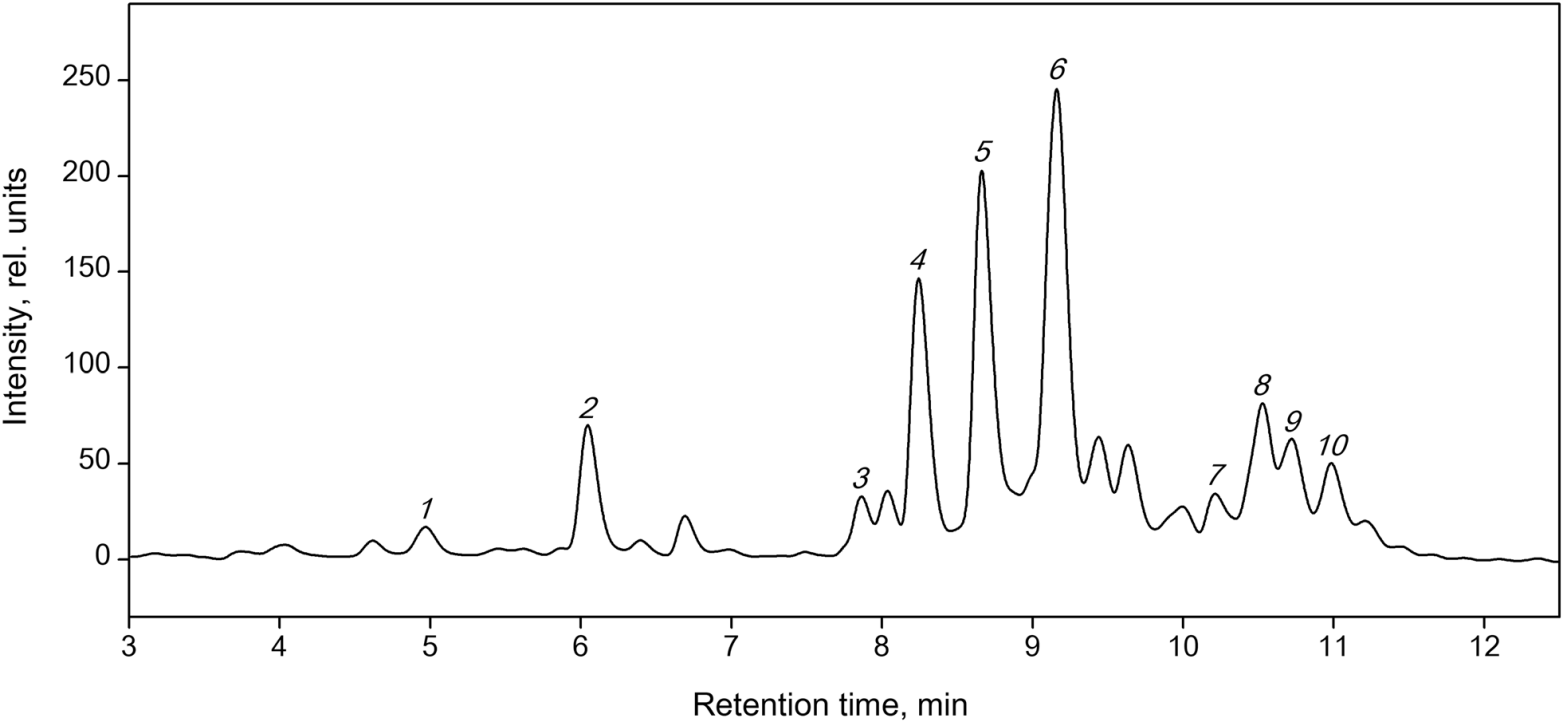
Typical chromatogram of the methanol extract of astaxanthin esters from *H. pluvialis* cells after 12 days of cultivation under stress conditions: (1) free astaxanthin; (2) lutein; (3)‒(6) monoesters of astaxanthin with hexadecatrienoic, linolenic, linoleic, and oleic fatty acid residues, respectively; (7), (9), (10) astaxanthin diesters; (8) β-carotene

**Fig. 6:**
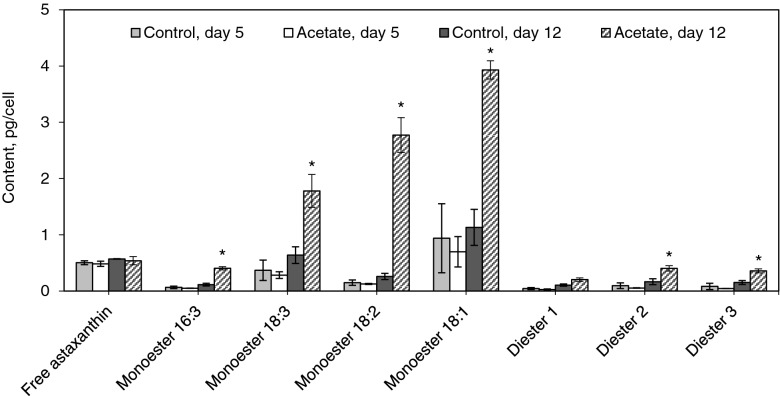
Content of astaxanthin and its fatty acid esters in *H. pluvialis* cells after 5 and 12 days of cultivation under stress conditions. Here, 16:3, 18:3, 18:2, and 18:1 indicates hexadecatrienoic, linolenic, linoleic, and oleic acid residues

The chromatogram obtained for methanolic extract of astaxanthin from *H. pluvialis* is shown in Fig. 5, and the results of calculations for the individual components are provided in Fig. 6. Note that no astaxanthin was detected at the beginning of the experiment (day 0) in the samples of all variants. On 12 days of the experiment, the astaxanthin fraction in the control contained a total of 65% monoesters, 12% diesters, and 22% free astaxanthin. In the acetate variant, where the formation of the cysts was more intense, the above-mentioned forms of astaxanthin amounted to 84%, 11%, and 6%, respectively. These findings approximately match the published data, which state that the extract from the red cysts of the microalga contains 70% monoesters, 25% diesters, and only 5% free astaxanthin (Zhekisheva et al. 2002; Solovchenko 2015).

During the exposure to high-intensity light, the content of astaxanthin esters slightly increased over time, whereas the content of the free astaxanthin did not depend significantly on the time or variant. On 5 days of the experiment, the contents of astaxanthin esters were not substantially different between the two variants. On 12 days of the experiment, the accumulation of astaxanthin mono- and diesters was especially pronounced in the acetate variant. Namely, the total content of monoesters was 4.3 times higher than in the control, and that of diesters was 3 times higher. In other words, the compounds, which predominantly accumulated, were monoesters. They contained the residues of the following fatty acids (in the order of decreased content): oleic, linoleic, linolenic, hexadecatrienoic. It is interesting that the esters of the fatty acids containing a smaller number of double bonds, on average, accumulated more intensely. On the whole, the total amount of the pigment esters increased from days 5 to 12 by a factor of 8 when sodium acetate was used and only by a factor of 1.5 in the control sample (without sodium acetate).

Thus, we have found that high-intensity light induces the accumulation of astaxanthin esters in *H. pluvialis* cells. The use of sodium acetate during the exposure to high-intensity light resulted in a significantly higher accumulation of the esters of this pigment, mostly in the form of monoesters. Apparently, this is due to the effect of sodium acetate both as a nutrient substrate (contributing to more intense biosynthesis of various compounds) and as a stressor (probably via the aforementioned mechanism of the change in the C/N ratio in the culture medium), and this fact is in agreement with an acceleration of the cyst formation process under these conditions.

### All-***E-*** and ***Z***-isomers of astaxanthin in ***H. pluvialis*** red cells

**Fig. 7:**
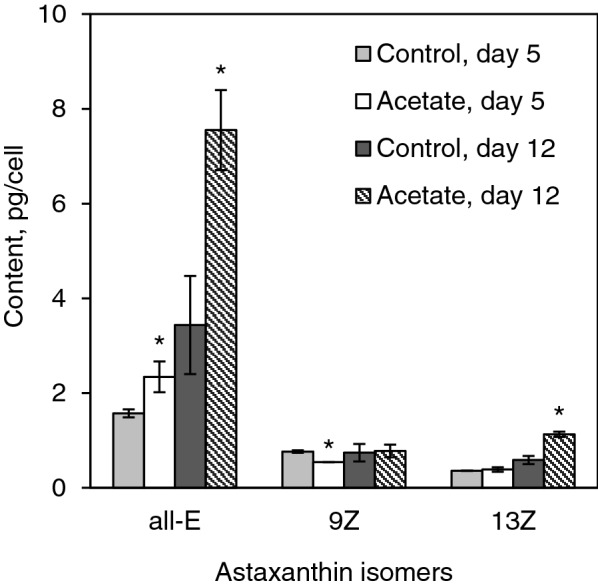
Content of specific astaxanthin isomers in *H. pluvialis* cells after 5 and 12 days of cultivation under stress conditions

According to the analysis of the saponified astaxanthin extracts (Fig. 7), the use of sodium acetate leads to an increase in the content of all-*E*-astaxanthin in the hydrolysate on days 5 and 12 and in the content of 13*Z*-astaxanthin on day 12 of the experiment in comparison with the control. Just as in the case with astaxanthin esters, no isomers of astaxanthin were detected in the cell extracts obtained at the beginning of the experiment (day 0). We also recorded a progressive increase in the astaxanthin content in the hydrolysates over time in both variants (control and acetate), apparently due to the increasing amounts of monoesters in the algal cells.

Interestingly, the relative content of both *Z*-isomers compared to total astaxanthin decreased from day 5 to day 12 (Table 1). It is known that astaxanthin is synthesized de novo in form of *E*-isomer, which means that at earlier stages of stress *Z*-isomers of astaxanthin were accumulated. It also should be noted that relative content of 13*Z*-astaxanthin stayed virtually the same during the experiment and did not change between variants. The dynamics of relative content of other isomers shows that it is 9*Z*-isomer that undergoes transformation to all-*E*-isomer in our experiments.

**Table 1:** Relative content of individual astaxanthin isomers in *H. pluvialis* cells compared to total astaxanthin, %

Isomer	Control, day 5	Acetate, day 5	Control, day 12	Acetate, day 12
*E*	58.2	71.6	72.1	79.8
9*Z*	28.4	16.6	15.6	8.2
13*Z*	13.4	11.8	12.3	11.9
9*Z* + 13*Z*	41.8	28.4	27.9	20.2

Thus, the exposure to light both in the absence and in the presence of sodium acetate causes the accumulation of astaxanthin isomers in *H.* *pluvialis* cells. These factors mostly affect the content of all-*E*-astaxanthin and, to a significantly lesser extent, 13*Z-*astaxanthin. Obtained data indicates that astaxanthin under these conditions is predominantly accumulated in the form of *E*-isomer.

### Content of photosynthetic carotenoids and chlorophylls in cells

Since the main place of ROS generation (Yu et al. 2015; Focsan et al. 2017) and their main target under the exposure of higher plants and photosynthetic algae to high-intensity light is the photosynthetic apparatus, it was important to analyze the content of photosynthetic pigments under these conditions.

**Fig. 8:**
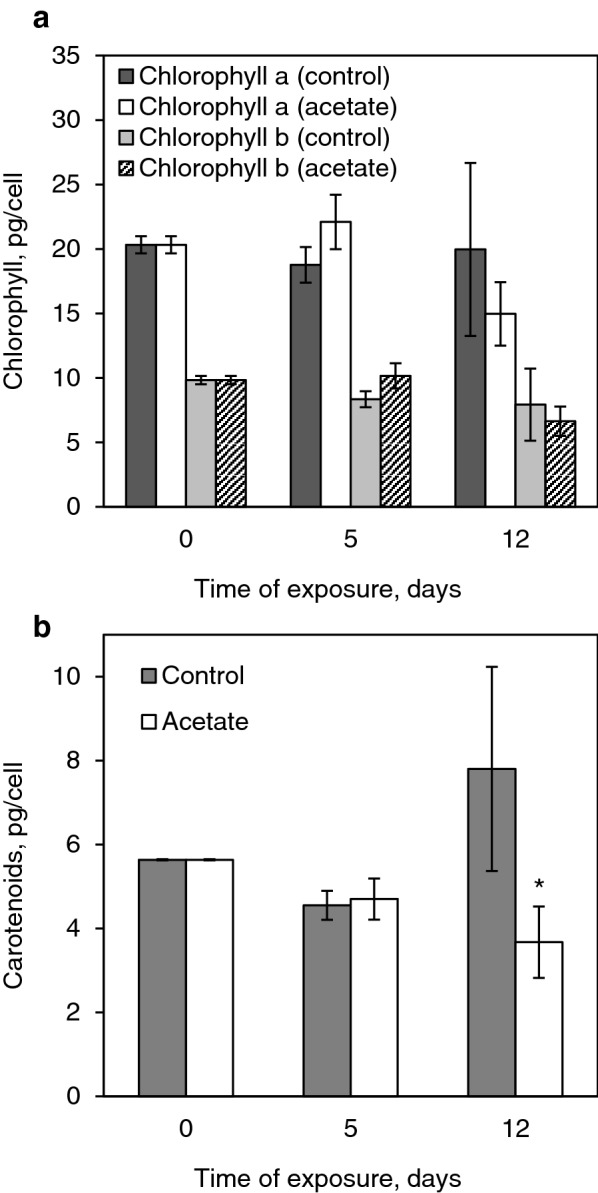
Content of photosynthetic pigments in *H. pluvialis* cells after 5 and 12 days of cultivation under stress conditions

The results of our analysis of the photosynthetic pigments are shown in Fig. 8. On the whole, no significant differences in the contents of chlorophylls were observed between different variants. At the same time, a slight decrease in the amount of chlorophylls *a* and *b* in the acetate variant in comparison with the control and with the initial values is found on day 12. It is interesting that the total content of photosynthetic carotenoids on day 12 is higher than the initial value in the control and, conversely, lower than the initial value if sodium acetate is used.

## Discussion

Presently astaxanthin is drawing increasing attention of researchers because of its protective effect during the development of various disorders (such as neurodegenerative and cardiovascular diseases) and its wide use in cosmetics and food products (Brustovetsky et al. 2005). Since both the free form of astaxanthin and its esters are used in the investigations, the aim of this research was to study the time pattern of the accumulation of astaxanthin mono- and diesters, as well as *Z*-isomers of astaxanthin in *H. pluvialis* cells experiencing different kinds of stress and to compare it to the process of *E*/*Z* isomerization of astaxanthin dissolved in an organic solvents under the action of high light intensity or heating.

There are abundant literature data on the conversion of the *E*-isomer of astaxanthin to the *Z*-isomer depending on the conditions (Yuan and Chen, 1999, 2001; Zhao et al. 2005; Chen et al. 2007; Lerfall and Birkeland, 2014) and just a few studies concerning the effect of light on the solutions of astaxanthin esters (de Bruijn et al. 2016; Honda et. al. 2020).

Summing up these results and ours, we can say that in a polar solvent under illumination at room temperature: the isomerization process is weak and total astaxanthin *Z*-isomers content is no more than 5%; the accumulation of 13*Z*-isomers significantly exceeds the accumulation of 9*Z*-isomers; under prolonged illumination, the content of both *Z*-isomers decreases due to oxidation and the formation of epoxy- and apo-products. The obtained results are in agreement with the data of (de Bruijn et al. 2016) and suggest that the *E*/*Z* isomerization under illumination, in contrast to the isomerization reaction in the dark, has an additional stage ‒ the formation of products of oxidative destruction. The reverse reaction of *Z*/*E* isomerization was recently investigated for astaxanthin under practically the same conditions as ours (illumination with light of similar intensity at temperatures of 30 °C and 50 °C) (Honda et al. 2020). However, the authors did not detect the formation of degradation and oxidation products. Possibly, this is due to fact that the spectra in the UV region, in which the degradation products absorbing light, were not studied.

Upon prolonged heating in an organic solvent in the dark, the degradation proceeds much slower, and the leading process is the accumulation of *Z*-isomers. According to the previously obtained data (Kulikov et al. 2020), the isomerization process depends on the dielectric constant of the medium, and a comparison of the percentage of isomers obtained by heating in chloroform and methanol (35% and 25%, respectively) confirms the previously established regularity. Noteworthy is the fact that the spectrum does not show the characteristic peak of apo- and epoxy-products at 250–260 nm, which may be associated with their further degradation during prolonged incubation at elevated temperatures. A decrease in the height of the main absorption maximum in this case can occur both due to the astaxanthin degradation and due to the accumulation of the 13*Z*-isomer, the extinction coefficient of which is lower than that of all-*E*-astaxanthin. Even if astaxanthin degradation takes place under such conditions, it seems to proceed much slower than the isomerization reaction; therefore, accumulation of *Z*-isomers is observed (see Fig. 3). Under illumination, the rate of oxidative destruction is high, and after 5 h it becomes higher than the rate of *Z*-isomers formation; therefore, a drop in absorption is observed.

As was mentioned before, astaxanthin is synthesized in algal cell in from of its all-*E*-isomer; therefore, all *Z*-isomer molecules detected in *H. pluvialis* cells must appear as a result of *E*/*Z* isomerization process. The mechanism of accumulation of *Z*-isomers in *H. pluvialis* cells in our experiments seems to be the light-induced *E*/*Z* isomerization. To the best of our knowledge, no enzymes were identified in the plant cell capable of catalyzing carotenoid *E*/*Z* isomerization, although CRTISO isomerase can catalyze the reverse reaction and can function in the dark (Isaacson et al. 2002; Yu et al. 2011). Heating was also not the case in our in vivo experiments. At day 5 of the illumination of algae cells, up to 42% of *Z*-isomers were detected in total astaxanthin pool. Upon further illumination, the summarized content of the *Z*-isomers decreased compared to the total astaxanthin content over time in similar way as in the organic solvent: the *Z*-isomers constituted 42% on day 5 but only 28% on day 12 in control. The same picture is observed if acetate is added: 28% on day 5 and 20% on day 12. Interestingly, although increasing total astaxanthin content, the addition of sodium acetate did not favor the formation of *Z*-isomers as much as the light alone, which makes it not useful for obtaining *H. pluvialis* biomass enriched with *Z*-astaxanthin. Apparently, while the accumulation of the *Z*-isomers at the initial stage of the experiment in a living cell, just as in the model system, is due to the process of photoinduced *E*/*Z* isomerization, the subsequent decrease of their fraction in the total astaxanthin pool is due to the oxidative degradation and/or reverse *Z*/*E* isomerization. Note that the role of light screening by this pigment increases as it is accumulated in the cell, and this can contribute to a decrease in the intensity of *E*/*Z* isomerization, while not impairing possible enzymatic reverse *Z*/*E* isomerization. At the same time, new all-*E*-isomer molecules are being continuously synthesized in a living *H. pluvialis* cell under such conditions*.*

We also assume that relatively high content of *Z*-isomers registered in in vivo experiments can be attributed, at least partly, to the fact that, in the cell, astaxanthin molecules are contained within the oil globules with a low dielectric constant (Ota et al. 2018), which favors the formation of *Z*-isomers (Yuan and Chen 1999; Kulikov et al. 2020), as was shown in the model system experiments. It is possible that a high content of *Z*-isomers, which must loosen the lipid packing, inhibits the energy transfer from the pigments of the light-harvesting antennae to the chlorophyll of the reactive centers of photosystems, thus protecting the photosynthetic apparatus under the conditions of overillumination.

Although experimental conditions were very different for in vitro and in vivo systems, the comparison of their results can help to understand the details of astaxanthin isomerization in algal cells. Such comparison leads us to conclusion that in both systems the astaxanthin *E*/*Z* isomerization process was defined by a) the action of light (or heat), and b) the dielectric constant of the surrounding medium.

A significant difference between model systems and red *H. pluvialis* cells is in the ratio of 9*Z*- and 13*Z*-isomers. Therefore, on the 5th day, the content of the 9*Z*-isomer was higher than that of the 13*Z-*isomer. The relative dynamics of 9*Z*- and 13*Z*-astaxanthin contents in our experiments was completely different from the one reported by Gong and co-authors: in their study, the relative content of 9*Z*-isomer was virtually the same after day 3 of experiment and further on while a slight increase in 13*Z*-isomer content was registered between days 6 and 12 (Gong et al. 2020). This may be associated with strain-specific differences in stress response.

The determination of the fatty acid composition of the monoesters showed that they contained the residues of the following fatty acids (in the order of decreasing content): oleic, linoleic, linolenic, and hexadecatrienoic. These findings show good agreement with the results of (Recht et al. 2014) and are exactly the opposite of the data of (Weesepoel et al. 2013), in which monoesters formed the following series according to their content: linolenic, linoleic, oleic. The differences in the fatty acid composition of the monoesters once more confirm the observation that the results obtained using living cells depend on the conditions of the experiment, mainly on the strain, composition of the incubation medium, light intensity, etc. This dependence is quite noticeable if we compare the contents of the 18:1 monoester in the media with and without sodium acetate: the contents of this monoester on day 12 differ almost by a factor of 4.

It is likely that the accumulation of astaxanthin and the formation of *H.* *pluvialis* cysts under the exposure to high-intensity light take place within the framework of the protective response, successful implementation of which ensures the protection of the photosynthetic apparatus of the algal cells, which is, in turn, will enable their normal function after the stressor is removed. As to the protective effect of astaxanthin with regards to photosynthetic chlorophyll, on the one hand, the content of chlorophyll usually decreases under astaxanthin-inducing conditions (Kakizono et al. 1992; Zhekisheva et al. 2002; Ota et al. 2018). On the other hand, astaxanthin can protect chlorophyll against high-intensity light in at least two ways: by screening the light and by neutralizing the emerging reactive oxygen species. The protective effect of astaxanthin is also confirmed by the data on the cell death when the astaxanthin synthesis is inhibited under such conditions (Fan et al. 1998). The data on the content of chlorophylls presented here can be explained by the efficient protective action of the astaxanthin esters, which are actively accumulated in the cells that have survived. At the same time, we have earlier shown a decrease in the content of photosynthetic pigments under similar conditions on day 20 of the experiment (Viazau et al. 2020). Presumably, 12 days of exposure to high-intensity light is insufficient for such an effect.

As is noted above, astaxanthin and its esters are absent in the green cells of the microalga; they are synthesized only under stress conditions. At the same time, the exposure to the stress causes most of the cells (80%) to die at the initial stage (5 days); during further exposure (12 days), the cells still die, but at a significantly lower rate. However, the addition of a nutrient source (sodium acetate) impedes this process. The influence of acetate is especially well demonstrated by the example of chlorophylls *a* and *b*, whose content on day 5 in the presence of acetate does not decrease and even increases to some extent. It is interesting that in this regime, in the presence of acetate, maximum ratio of the *E*-isomers of astaxanthin is observed on day 12. It should be noted that sodium acetate is a nutrient substrate, which boosts the growth rate of green cells. Nevertheless, it is assumed that it can have a stress effect due to the change in the C/N ratio in the culture medium, which is perceived by the cell as nitrogen deficiency (Kakizono et al. 1992; Kobayashi et al. 1993).

In *H. pluvialis* cells exposed to high-intensity light, the content of astaxanthin esters increased over time, while free astaxanthin content virtually did not change. This fact assumes the increased biosynthesis of the pigment with subsequent formation of its esters with fatty acids, matching the existing concept of astaxanthin accumulation in *H. pluvialis* cells affected by stressors. The monoesters are the main fraction of the astaxanthin in this case, and it is their properties (rather than the free form) that must determine the functions of the astaxanthin. However, their content in the cell is also important. As follows from the results at the initial stage, where the content of all the forms of the astaxanthin is low, they cannot protect the majority of the cell from overillumination. Only when their content increases by day 12, especially that of 18:1 monoesters, the cells cease to die. One can assume that the high content of the *Z*-isomers is necessary to transform part of the energy of the absorbed light quanta into heat, a process that can prevent the damage to the function of the photosynthetic apparatus under the conditions of overillumination and intense accumulation of reactive oxygen species.

## Conclusion

The study of the photoinduced astaxanthin isomerization both in the model system in vitro and in living *H. pluvialis* cells shows the general trend of this process over time: first, the *Z*-isomers are accumulated, and then their part in the total astaxanthin pool decreases. In the cells, the relative content of astaxanthin *Z*-isomers at the initial stages of the experiment was substantially higher than that in the model system. The drastic decrease in *Z*-astaxanthin content during the time of the experiment in this case can probably be explained by an increasing role of such factors as light screening by astaxanthin itself, oxidative degradation of the pigment and de novo all-*E*-astaxanthin synthesis.

Our findings suggest that it is possible to choose the conditions for cell cultivation depending on the goal: if it is necessary to increase the total yield of astaxanthin and its esters, then long-term illumination in the presence of sodium acetate is instrumental; if an increased content of *Z*-isomers is required, the cells should be illuminated for a short period of time without sodium acetate. Although heating of astaxanthin extracts allows to achieve ratio of *Z*-isomers similar to the highest detected in living cells, the cultivation of *H. pluvialis* culture in conditions favorable for *Z*-astaxanthin accumulation still can be considered highly promising method for obtaining large amounts of *Z*-astaxanthin (possibly in combination with further heat treatment of extracts from the biomass), which has more prominent antioxidant properties and health benefits, better bioavailability and extractability compared to all-*E*-astaxanthin.

Further research, focusing on increasing biomass production and total astaxanthin production, is needed for the application of our results in biotechnological process of obtaining *H. pluvialis* biomass enriched with *Z*-astaxanthin.

### Supplementary Information

**Additional file 1.** Supporting data (spectra, chromatogram).

## Data Availability

The authors declare that the data supporting the findings of this study are available within the article, or are available upon reasonable requests to the authors.

## Abbreviations

*H. pluvialis*: *Haematococcus pluvialis*
HPLC: High-performance liquid chromatography
MS: Mass spectrometry
