# Supplementary material for: E/Z isomerization of astaxanthin and its monoesters in vitro under the exposure to light or heat and in overilluminated Haematococcus pluvialis cells
Source: Bioresour Bioprocess. 2021 Jul 1;8(1):55. doi: 10.1186/s40643-021-00410-5 (PMC10992054; doi:10.1186/s40643-021-00410-5)
Supplement: Supplementary file 1 — Additional file 1. Supporting data (spectra, chromatogram). [file 40643_2021_410_MOESM1_ESM.docx]

**Supplementary material**


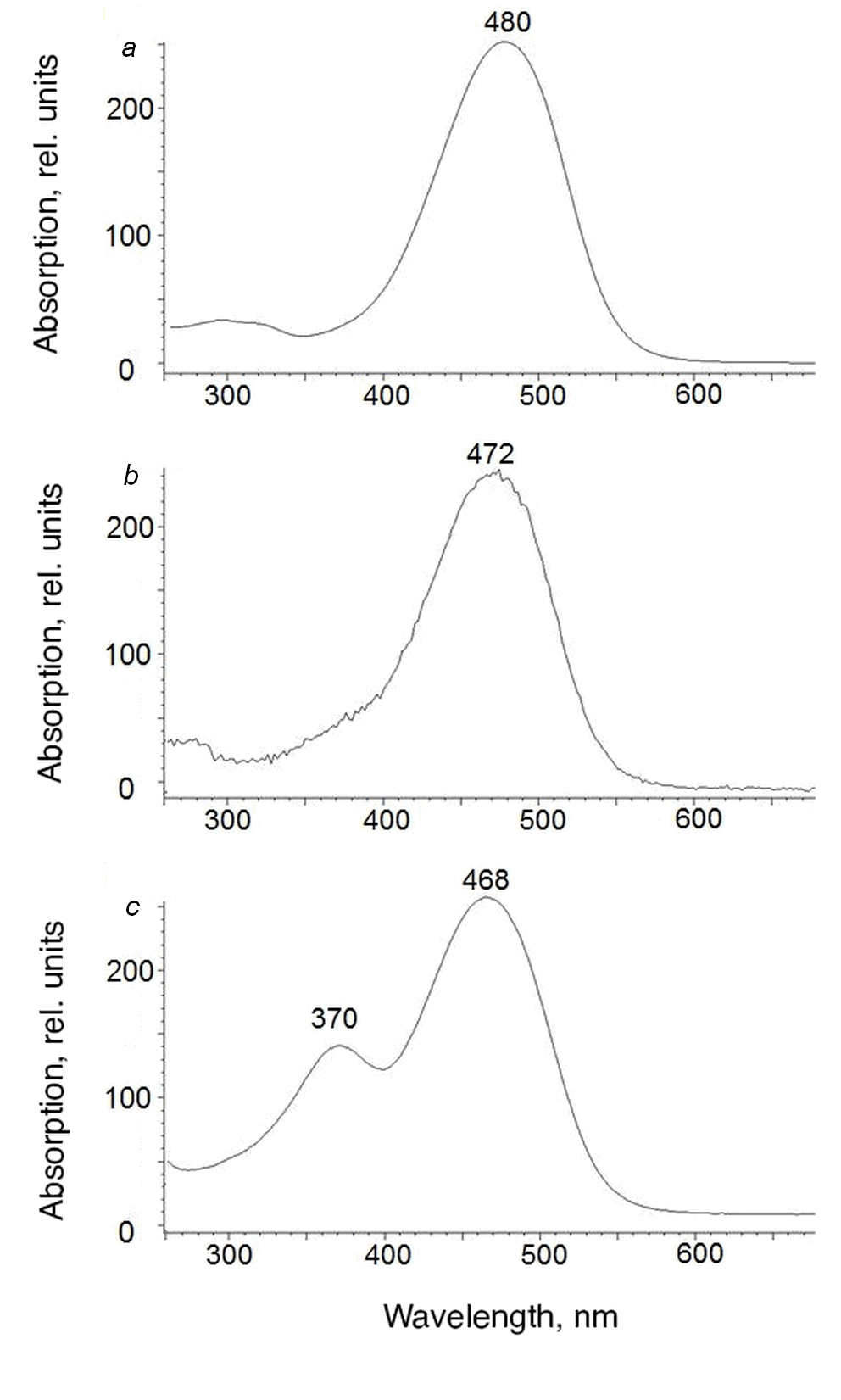


**Fig. S1** Absorption spectra for the chromatogram peaks 1–4: (А) all-*E*-astaxanthin, (B) 9*Z*-astaxanthin, (C) 13*Z*-astaxanthin. The solvent is the same as the mobile chromatographic phase: МeOH : CH_2_Cl_2_ : CH_3_CN : H_2_O = 82.5 : 5 : 5.5 : 7

**Fig. S2** Absorption spectra of astaxanthin solution in methanol before isomerization, after irradiation for 5 hours and after incubation at 40°С for 4 days


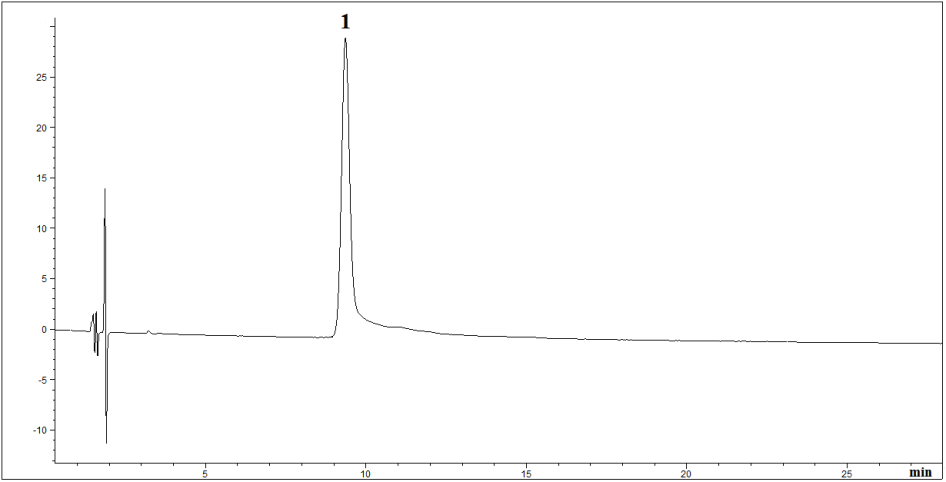


**Fig. S3** Chromatogram of all-*E*-astaxanthin solution used in the experiments, in methanol before isomerization


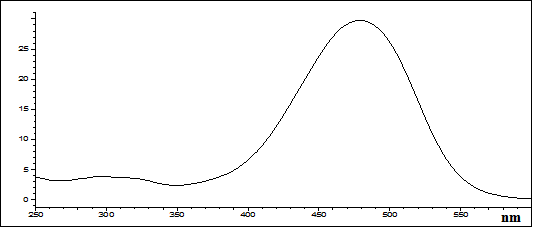


**Fig. S4** Light absorption spectrum for Peak 1 at chromatogram of all-*E*-astaxanthin solution, corresponding to pure all-*E*-astaxanthin
